# The Australian Pharmaceutical Benefits Scheme data collection: a practical guide for researchers

**DOI:** 10.1186/s13104-015-1616-8

**Published:** 2015-11-02

**Authors:** Leigh Mellish, Emily A. Karanges, Melisa J. Litchfield, Andrea L. Schaffer, Bianca Blanch, Benjamin J. Daniels, Alicia Segrave, Sallie-Anne Pearson

**Affiliations:** Pharmacoepidemiology and Pharmaceutical Policy Research Group, Faculty of Pharmacy, University of Sydney, A15, Pharmacy and Bank Building, Sydney, 2006 Australia; Drug Utilisation Section, Pharmaceutical Benefits Division, Department of Health, Canberra, 2601 Australia; Centre for Big Data Research in Health (CBDRH), University of NSW, Level 1, AGSM Building (G27), Sydney, 2052 Australia

**Keywords:** Pharmacoepidemiology, Drug prescriptions, Drug utilisation, Databases, Australia

## Abstract

**Background:**

The Pharmaceutical Benefits Scheme (PBS) is Australia’s national drug subsidy program. This paper provides a practical guide to researchers using PBS data to examine prescribed medicine use.

**Findings:**

Excerpts of the PBS data collection are available in a variety of formats. We describe the core components of four publicly available extracts (the Australian Statistics on Medicines, PBS statistics online, section 85 extract, under co-payment extract). We also detail common analytical challenges and key issues regarding the interpretation of utilisation using the PBS collection and its various extracts.

**Conclusions:**

Research using routinely collected data is increasing internationally. PBS data are a valuable resource for Australian pharmacoepidemiological and pharmaceutical policy research. A detailed knowledge of the PBS, the nuances of data capture, and the extracts available for research purposes are necessary to ensure robust methodology, interpretation, and translation of study findings into policy and practice.

## Findings

In recent decades the discipline of pharmacoepidemiology has experienced exponential growth, both in Australia [1] and internationally [2–4]. This growth has been driven, at least in part, by the increasing availability of routinely collected dispensing databases and increased access to data linkage of dispensing claims and other health data collections. In addition, there has been recognition of the limitations of randomised controlled trials (RCTs) to inform post-market medicine use, including their short duration, small sample size, and non-representative patient populations [5]. Studies using routinely collected databases overcome many of these difficulties, complementing findings from RCTs and offering valuable insights into the real-world use, safety, and effectiveness of medicines [2].

There are a wealth of databases available for pharmacoepidemiological research, and while there are commonalities across health jurisdictions, each dataset has its own idiosyncrasies with respect to analysis and interpretation. Researchers must be familiar with the limitations and features of their chosen dataset. The purpose of this paper is to provide a practical guide for researchers using Australia’s Pharmaceutical Benefits Scheme (PBS) dispensing data. Specifically, we will set the scene by describing the PBS, the Australian Government’s subsidised prescription medicines program. We will then detail the specifics of the PBS data collection and the PBS data extracts available to examine trends in prescribed medicine use. Finally, we will consider some of the factors affecting the interpretation of prescribed medicine use based on PBS claims.

## Background: the Pharmaceutical Benefits Scheme (PBS) and Repatriation Pharmaceutical Benefits Scheme (RPBS)

Australia has universal health care arrangements entitling all citizens and permanent residents to a range of subsidised healthcare services. Prescription medicines are subsidised under two Commonwealth schemes: the PBS and the Repatriation Pharmaceutical Benefits Scheme (RPBS). The PBS subsidises prescribed medicines for Australian residents and eligible foreign visitors (i.e. those with reciprocal health care agreements with Australia). The RPBS, available to eligible war veterans and their families, comprises all PBS-listed medicines and additional subsidised pharmaceutical items [6].

The Australian Government reimburses community pharmacies and private hospitals for PBS-listed medicines, subsidising approximately 75 % of prescribed medicine use in Australia [7]. Prescriptions dispensed to public hospital inpatients have not traditionally been PBS-subsidised; however, the Australian Government has individual agreements with most Australian states and territories (except New South Wales and the Australian Capital Territory) under the Public Hospital Pharmaceutical Reforms, enabling participating hospitals to provide discharging patients and outpatients with PBS-subsidised medicines [8].

### Schedule of Pharmaceutical Benefits

The PBS underpins Australia’s National Medicines Policy, which is concerned with providing access to safe, effective, and affordable medicines, and ensuring their quality use [9]. The PBS is governed by the *National Health Act 1953* and the *National Health* (*Pharmaceutical Benefits*) *Regulations 1960* (*Cth*) *Act*, and is administered by the Department of Human Services (DHS; formerly known as Medicare Australia and the Health Insurance Commission) under the *Health Insurance Act 1973* (*Cth*).

A medicine is eligible for listing on the PBS or RPBS after it has been registered for use in Australia by the Therapeutic Goods Administration (TGA). To gain listing it must be assessed and recommended by the Pharmaceutical Benefits Advisory Committee (PBAC), an independent expert body appointed by the Australian Government. In making its recommendation, PBAC considers the medicine’s clinical place, effectiveness, safety, cost, and cost-effectiveness compared to currently available treatments [10]. After a positive recommendation from PBAC, the Minister for Health authorises the medicine to be listed on the Schedule of Pharmaceutical Benefits.

### PBS benefit categories

Medicines are PBS-listed according to one of three benefit categories [11]:

- *Unrestricted benefits* Medicines available for general use without limits on the subsidised indication for prescribing.
- *Restricted benefits* Medicines available for the treatment of certain indications or patient groups. If the medicine is prescribed outside the PBS-specified indication, prescribers are required to write private (unsubsidised) prescriptions.
- *Authority required benefits* An authority prescription is required for certain restricted medicines and for cases where a higher dose or quantity of the medicine is required than the maximum approved on the PBS. Authority benefits fall into two categories: (a) *Authority required* prescriptions, which require the prescriber to obtain written or telephone approval from DHS or Department of Veterans’ Affairs (DVA) before dispensing is permitted; and (b) *Authority required* (*STREAMLINED*) prescriptions, which do not require prior approval from DHS or DVA, but a streamlined authority code must be provided on the prescription [12].

### Sections of the Schedule of Pharmaceutical Benefits

Medicines listed on the Schedule of Pharmaceutical Benefits are also classified into sections based on the *National Health Act 1953* [6, 13, 14].

- Section 85 (s85) of the *Act* pertains to medicines available under the *standard arrangements* established for medicine subsidy. S85 or *general* medicines comprise the majority of prescriptions supplied under the PBS and RPBS.
- Section 100 (s100) refers to medicines subsidised under *special arrangements*. For example, s100 medicines may be restricted to supply through a hospital with specified specialist facilities [15]. This section includes many specialty, high cost medicines, such as chemotherapy and chronic illness agents, as well as medicine programs including the Botulinum Program, Growth Hormone Program, In Vitro Fertilisation/Gamete Intrafallopian Transfer Program, and the Opiate Dependence Treatment Program [6, 14]. Special arrangements also exist for the supply of PBS medicines to clients of eligible Aboriginal Health Services in remote areas of Australia [16].

### Patient categories

#### Beneficiary status

To ensure affordability of medicines for all Australians, the level of subsidy under the PBS depends on the patient’s beneficiary status. Concessional status was established for individuals who are eligible to receive government entitlements, including pensioners, low-income earners, repatriates, and Indigenous Australians living with or at risk of chronic illness (the latter under the Closing the Gap co-payment measure) [17, 18]. Eligible veterans and their dependents holding a DVA health card are also entitled to medicines and additional pharmaceutical items at concessional rates under the RPBS [19]. These patients have a low co-payment threshold. All other individuals are considered general beneficiaries and have a higher co-payment threshold. As of 1 January 2015, the maximum co-payment was AUD$6.10 for concessional beneficiaries and AUD$37.70 for general beneficiaries [20]. For medicines costing more than the relevant beneficiary co-payment (i.e. over co-payment), additional costs are paid by the Commonwealth. Medicines costing less than the relevant co-payment (i.e. under co-payment) are not subsidised, but paid in full by the patient. Currently all PBS-listed medicines are priced above the concessional beneficiary co-payment but may be priced above or below the general beneficiary co-payment.

#### PBS Safety Net

The PBS Safety Net was established to provide financial assistance to individuals and their families spending large amounts on medicines in a calendar year. Once a family collectively spends over the threshold amount (as of 1 January 2015, AUD $1453.90 for general beneficiaries and AUD $366.00 for concessional beneficiaries), the subsequent cost of dispensings for all family members are reduced such that general beneficiaries pay the concessional co-payment rate, and concessional beneficiaries have no co-payments so they receive medicines free of charge [20]. As the reduced cost of dispensings can encourage patients to obtain additional quantities of medicines before they are needed, the Government introduced the Safety Net 20 day rule on 1 January 2006 to encourage responsible use of the PBS. The rule means that, for selected PBS-subsidised medicines used for long-term therapy, a repeat supply of the same medicine within 20 days does not count towards reaching the PBS Safety Net threshold. Moreover, these medicines will not be supplied at the reduced price if the threshold has already been reached [21].

#### Doctor/prescriber bag (emergency drug supply)

Certain PBS medicines are provided to doctors to treat patients in emergencies [22]. These medicines are free for the doctor and patient as they are completely subsidised by the Commonwealth.

### The PBS/RPBS data collection

PBS/RPBS dispensing claims submitted for payment of a government subsidy are processed by DHS and provided to the Department of Health (DoH) and DVA (for their clients only) for monitoring, evaluation, and health service planning.

As of 1 April 2012, DHS also processed dispensing records for under co-payment medicines. The collection of under co-payment records was agreed to under the *Fifth Community Pharmacy Agreement* and legislated under the *National Health Amendment* (*PBS*) *Act 2010* [23]. As a result, under co-payment dispensing data are now recorded in the PBS data collection.

When a PBS/RPBS medicine is dispensed, the administering pharmacy or hospital provides DHS with data pertaining to the prescription dispensed, identity of the patient, prescribing doctor, and supplying pharmacy. This information forms the basis of the PBS collection. Identifying information about the patient, doctor and pharmacy (such as names and addresses) is not available to researchers or in the public domain. Moreover, policies such as the suppression of cell counts in aggregated public domain data also apply to protect patient privacy. A summary of key variables provided in the PBS collection is provided in Table 1.

**Table 1:** Core variables present in the PBS data collection. Availability to researchers depends on the data extract

Variable	Definition
Medicine details	
ATC code	Internationally accepted, WHO-defined codes^a^ that classify medicines over five levels, starting broadly with the anatomical site of action (e.g. nervous system) and ending specifically with the chemical substance (e.g. oxycodone) [44]
PBS item code	Pharmaceutical Benefits Scheme defined codes that provide medicine details at the product level, including generic name, form, strength, administration route, quantity per unit (pack size), and approved indication, where applicable
Medicine section	Classification according to section of the PBS Schedule (section 85 or 100)
Prescription details	
Date of prescription	Date on which the prescription was written
Date of supply	Date on which the medicine was supplied/dispensed by the pharmacy or hospital
Date of processing	Date on which the claim was processed by DHS
Prescription type	Describes whether the prescription is an original, repeat, deferred supply, authority, etc
Total cost	The gross price of the prescription, including the patient contribution plus the net benefit
Patient contribution	The amount paid by the patient for the prescription
Government contribution	The benefit paid to the pharmacy by the Australian Government
Prescription category	The program under which the prescription was dispensed (e.g. PBS, RPBS, under co-payment, private etc.)
Regulation 24 status	Indicates that the original supply and all repeats were dispensed at once
Streamlined authority code	Indicates the physician-declared indication or reason for prescription for *Authority required* (*STREAMLINED*) medicines
Patient details	
Patient identifier	A unique, scrambled patient identifier provided by the Australian Government, allowing derivation of additional patient characteristics such as age (via date of birth), sex and geographical location
Patient category	The beneficiary status of the patient (e.g. concessional, general, safety net, doctor's bag, under co-payment, Closing the Gap); determines how much the patient contributes to their medicine cost
Patient location	The location (e.g. state, statistical local area) of the patient
Measures of utilisation	
Quantity	The quantity of medicine supplied to the patient
Number of dispensings/scripts	The number of prescriptions dispensed (including original and repeat)
DDD/1000 pop/day	A measure of utilisation based around the WHO Defined Daily Dose (DDD), allowing for standardisation of use across different countries and drug formulations; provides a rough estimate of the proportion of the population treated daily with the medicine of interest [45]
Prescriber information	
Prescriber identifier	A unique, scrambled number identifying the prescribing doctor
Prescriber specialty	Identifies the specialty of the prescribing doctor (e.g. general practitioner, psychiatrist etc.)
Prescriber location	The location (e.g. state, statistical local area) of the prescribing doctor
Pharmacy information	
Pharmacy identifier	A unique, scrambled number identifying the dispensing pharmacy
Pharmacy location	The location (e.g. state) of the dispensing pharmacy

*ATC* Anatomical Therapeutic Chemical, *DDD*/1000 *pop*/*day* defined daily dose per 1000 population per day, *DHS* Department of Human Services, *WHO* World Health Organisation

^a^ATC codes provided in the PBS dataset may occasionally differ from those determined by WHO

Details of medicine dispensings requiring written or telephone authorisation are stored in a separate Authority Approvals database by DHS. This database records prescription details for *authority required* medicines, including patient identifiers, PBS item code, the date on which authority approval was obtained, prescriber identifiers, and the authority restriction number, which provides the reason, or indication, for dispensing as declared by the prescribing physician. However, the validity of authority codes (including streamlined) for inferring patient diagnosis is uncertain; this relies on the doctor or pharmacist selecting the correct code.

### PBS/RPBS data extracts

PBS/RPBS data are available in a variety of formats. Aggregated (de-identified) data are available online from DHS [24] or DoH [25] in fixed or interactive forms, while more detailed, customised reports in aggregated or unit-record formats can be requested from DHS, DoH, or DVA.

#### Publicly available data

##### Australian Statistics on Medicines (ASM)

The ASM reports are produced annually by the Drug Utilisation Sub-Committee (DUSC) of the PBAC for the purpose of estimating total community use of prescription medicines based on volume, defined daily dose (DDD) per 1000 population per day (DDD/1000 pop/day), and government and patient costs [26]. The DUSC database, which is the source of the ASM reports, combines the PBS/RPBS dataset with estimates of non-subsidised (under co-payment and private) prescription medicines use obtained from an ongoing Pharmacy Guild Survey of approximately 370 community pharmacies. However, the Pharmacy Guild Survey ceased on 1 August 2012 with the establishment of the *Fifth Community Pharmacy Agreement* and subsequent collection of under co-payment dispensings by DHS. ASM reports are presented according to PBS item and Anatomical Therapeutic Chemical (ATC) codes, aggregated by year of dispensing and according to the recording source (PBS/RPBS or Pharmacy Guild Survey). The ASM is available in print from 1991 to 1996, and online from 1997 [26].

##### Medicare Australia PBS item and group reports (hereafter PBS Statistics online)

DHS has developed online, interactive reports detailing aggregated PBS and RPBS dispensing volume and costs to government [24]. The customised reports, available from 1992, can be downloaded based on individual PBS item codes, or grouped by ATC classifications (e.g. alimentary tract and metabolism) or patient category. Reports can also be formatted according to the state/territory location of the dispensing pharmacy, and according to month, calendar year or financial year of processing. Importantly, the reports do not include data on under co-payment or private prescriptions and there is limited capture of s100 medicines [27]. Data on s100 highly specialised drugs dispensed in public hospitals are not available prior to July 2013; a transition to online claiming in public hospitals enabled collection of data at the level of the individual prescription from this date [28]. Reports are based on date of processing by DHS, not the date of supply.

##### Section 85 extract

DoH has developed an online, downloadable extract of s85 dispensing records [25]. Reports are available by month of processing (annually from 2008/2009, with monthly updates), or month of supply (single extract from July 2009, excluding most recent 6 months). Both reports aggregate volume and costs according to patient category. These data also include under co-payment medicines from 1 July 2012 and Closing the Gap under co-payment data from 1 July 2010.

##### PBS/RPBS under co-payment extract

An online, downloadable extract of PBS/RPBS under co-payment data was made available by DoH in July 2012 [29]. The extract is based on date of processing and aggregates volume by month, according to patient category, scheme (PBS, RPBS), and schedule (s85 or s100).

#### Data available on request

DHS, DoH, and DVA have mechanisms in place for researchers to request PBS/RPBS reports in aggregated or unit-record format to address specific research questions. Customised extracts of the DUSC database can be requested from the PBS Information Management Section, Pharmaceutical Policy Branch, Department of Health, and are provided in aggregated (de-identified form) from 1987, incorporating the Pharmacy Guild Survey from 1989 to August 2012. After this period the DUSC database extracts incorporate PBS/RPBS and under co-payment dispensings. These extracts have the advantage of more complete medicines capture than the PBS/RPBS databases alone. Another PBS extract worth mentioning is the PBS 10 % sample, a standardised, longitudinal, unit-record extract containing all PBS medicine dispensing data for a random 10 % sample of Australians. Access is established via a contract with DHS. Researchers can also obtain access to a Fact of Death Data (FODD) file from DHS. This file is compiled by the Australian Institute of Health and Welfare using monthly data from state and territory registries of births, deaths and marriages, and is matched to PBS/RPBS dispensing records [30]. All other data extracts require specific approval.

The main features of the data extracts in this section are described in Table 2.

**Table 2:** Comparison of data extracts: information available to researchers

	ASM	DUSC	PBS Statistics online	PBS Section 85	Under co-payment	PBS 10 % sample
Access						
Online	✓		✓	✓	✓	
By request		✓				✓
Data custodian	DoH	DoH	DHS	DoH	DoH	DHS
Access fee		✓				✓
Level of record						
Aggregate	✓	✓	✓	✓	✓	
Unit-level						✓
Date of record						
Date of processing			✓	✓	✓	✓
Date of supply	✓	✓		✓		✓
Date of prescription						From Sep 2012
Time						
Data available (start–end)	1991–2011	1987–present	1992–present	2009–present	2012–present	2005–present
Measurement unit^a^	Year	Month	Month	Month	Month	Day
Frequency of updates	Calendar year	By request	Month	Month	Financial year	Quarter
Patient information						
Scrambled identifier						✓
Age^a^		5 year age groups^b^				From year of birth
Sex		✓^b^				✓
Geographical area^a^	National	State^b^	State	National	National	National
Fact of death						Year of death
Prescriber information						
Scrambled identifier						✓
Specialty		✓^b^				✓
Pharmacy information						
Geographical location			State			State
Medicine classification						
ATC code	✓	✓	Highest level only	✓		
PBS item code	✓	✓	✓	✓	2012–2013 report only	✓
Patient category						
General beneficiary		✓^b^	✓	✓	2012–2013 report only	✓
Concessional beneficiary		✓^b^	✓	✓	2012–2013 report only	✓
Repatriation beneficiary		✓^b^	✓	✓	2012–2013 report only	
‘Closing the Gap’		✓^b^		✓		
Doctor’s bag		✓^b^	✓	✓		✓
PBS dispensing data capture						
PBS	✓	✓	✓	✓	2013–2014 report only	✓
RPBS	✓	✓	✓	✓	2013–2014 report only	
Additional dispensing capture						
Under co-payment dispensing	From 1989	From 1989		From 2012	From 2012	
Private dispensing	1989–2011	1989–2012				
Medicine sections						
Section 85	✓	✓	✓	✓	✓	✓
Section 100	Limited	Limited	Limited		✓	✓
Measures						
Volume (no. of dispensing records)	✓	✓	✓	✓	✓	✓
DDD/1000 population/day	✓	✓				
Cost to patient		✓^b^		✓		
Cost to government		✓^b^	✓	✓		
Total cost (patient + government)	✓	✓^b^		✓		

Information accurate as of 1 July 2015

*ASM* Australian Statistics on Medicines, *ATC* Anatomical Therapeutic Chemical, *DHS* Department of Human Services, *DoH* Department of Health, *DUSC* Drug Utilisation Sub-Committee, *PBS* Pharmaceutical Benefits Scheme, *RPBS* Repatriation Pharmaceutical Benefits Scheme

^a^Smallest unit available

^b^Available for PBS/RPBS data only

## Factors affecting the interpretation of prescribed medicine use based on PBS/RPBS claims

This section describes some of the common challenges encountered when using the PBS/RPBS database and its various extracts to obtain utilisation estimates.

### Seasonality

**Fig. 1:**
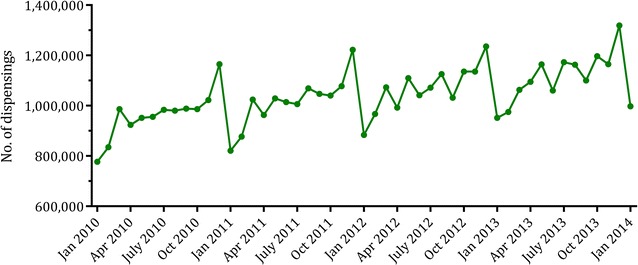
The seasonality effect of dispensing records. Monthly dispensings of proton pump inhibitors to concessional beneficiaries, January 2010 to January 2014. Pharmaceutical Benefits Scheme and Repatriation Pharmaceutical Benefits Scheme Section 85 Supply data, Australian Government Department of Health [25]

PBS data are subject to seasonality due to the effect of the Safety Net (Fig. 1). As previously mentioned, when a family spends over a specified amount on PBS medicines in one calendar year (i.e. exceeds the Safety Net threshold), the cost of all subsequent PBS medicines are reduced to the concessional rate for general beneficiaries and are free for concessional beneficiaries. This reduced medicine price on reaching the Safety Net can lead to a phenomenon known as *stockpiling*: Safety Net entitlements result in some patients obtaining extra quantities of their medicines toward the end of the year, stockpiling for the new year when they revert back to paying standard prices. This results in increased rates of dispensing of the medicine toward the end of the year followed by a trough at the start of the next year. Despite attempts to reduce this phenomenon through the introduction of the Safety Net 20 day rule on 1 January 2006, stockpiling continues to result in pronounced seasonality in utilisation data based on date of supply.

### Date of supply vs. date of processing

Each dispensing claim records the date a medicine is supplied to a patient by the dispensing pharmacy or the date the dispensing pharmacy’s claim for reimbursement is processed by DHS. Some of the data extracts include both of these variables while others include only one. For example, ASM reports are based around date of supply, PBS Statistics online use date of processing, and Section 85 online extracts are available by either date. There is often a discrepancy in utilisation measures based on date of supply versus those based on date of processing as the processing of the claim occurs some time after the prescription is dispensed and the interval of time between dispensing and processing is variable [25]. As such, caution must be employed when using data based around date of processing, particularly when examining medicine use at particular time points; these figures are primarily useful for obtaining rough approximations of utilisation. Date of supply should be used preferentially for examining medicine use.

**Fig. 2:**
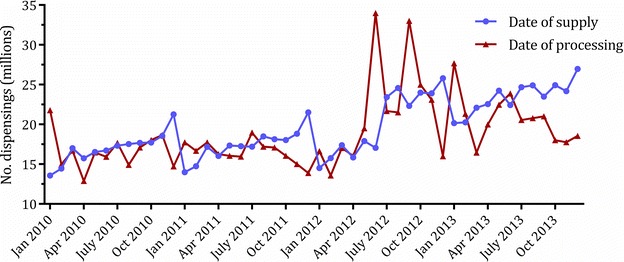
Number of dispensings by date of supply and date of processing. Monthly number of dispensings for all section 85 PBS-subsidised medicines January 2010 to January 2014, represented by date of supply and date of processing. Pharmaceutical Benefits Scheme and Repatriation Pharmaceutical Benefits Scheme Section 85 Supply and Processing Data, Australian Government Department of Health [25]

Figure 2 depicts the discrepancy between utilisation estimates based on date of supply compared to date of processing for all s85 PBS-subsidised medicines. Troughs in utilisation according to date of processing can be seen around late 2011 and late 2013, indicating delays in the processing of claims by DHS. The 2011 trough is followed by compensatory peaks, indicating a period of increased processing. Note that the data by date of supply show seasonal fluctuations as mentioned above. Seasonal fluctuations are less apparent in date of processing graphs due to the variable delay between dispensing and processing.

The risks of using date of processing data are well demonstrated by a recent example. In October 2013, the Australian Broadcasting Corporation’s *Catalyst* program aired a two part series questioning the link between high cholesterol levels and heart disease and suggested that the benefit of statins for preventing cardiovascular disease had been exaggerated. Much public debate followed the program. In May 2014, *Australian Doctor* published an article based on PBS statistics online data, reporting that there had been no change in statin dispensing for up to 3 months after the program aired [31]. However, our interrupted time series analysis using date of supply showed that there was an immediate and sustained 2.6 % reduction (equating to 500,000 fewer prescriptions) in statin dispensing persisting up to 8 months after the program aired [32].

One caveat concerning use of date of supply data is that dispensing records are not available in the dataset until the claim has been processed by DHS. Due to the variable delay in processing, this can result in incomplete ascertainment of claims dispensed on a given date for a number of months. It is therefore advisable to truncate the observation end date by at least 3 months, and preferably 6 months, to avoid under-reporting of utilisation. Indeed, the Section 85 Date of Supply report does not contain data for the most recent 6 months to ensure that the data are of a satisfactory level of completion before publication.

### Ascertainment and under co-payment medicines capture

As previously mentioned, the PBS database did not capture data on the dispensing of under co-payment medicines until at least April 2012 (July 2012 for Section 85 Date of Supply), thereby under-ascertaining the utilisation of certain medicines prior to this time. As under co-payment prescriptions comprised approximately 18 % of medicine use in 2011 [7], this issue significantly impacts utilisation estimates for certain drugs.

To determine if a particular PBS item was under-ascertained it is necessary to track the time points at which the cost of the medicine fell below the general co-payment threshold in the study period (prior to April 2012). As the co-payment threshold increases yearly and medicine prices change over time, the medicine’s price must be compared to the yearly co-payment threshold throughout the period of interest. The inclusion of under co-payment data in 2012 also must be considered when examining utilisation of under co-payment medicines over this period. It should also be noted that while DHS now records all under co-payment dispensing claims, not all of the collections detailed in this document incorporate under co-payment data as part of the extract (see Table 2).

**Fig. 3:**
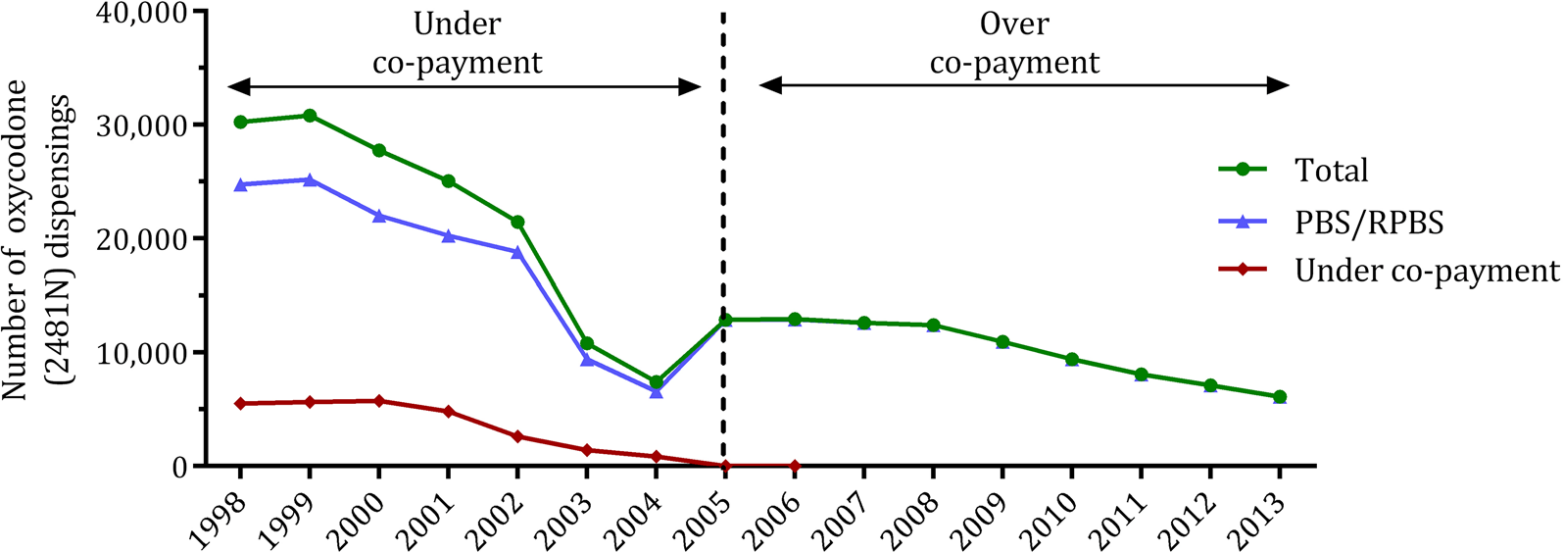
The effect of co-payment status on utilisation estimates, by script type. Yearly number of dispensings of oxycodone suppositories (30 mg; PBS item code 2481 N), 1998–2013. The co-payment status of the medicine is indicated. Note that measures of utilisation using PBS/RPBS prescriptions under-estimate total use when the medicine is under co-payment. Drug Utilisation Sub-Committee combined dataset, Australian Government Department of Health

An example of how these issues affect the data is provided in Fig. 3. Oxycodone suppositories (30 mg; PBS item code 2481N) were under co-payment between January 1998 and December 2004. Oxycodone utilisation was under-ascertained in the PBS dataset for the duration of this under co-payment period; only use by concessional beneficiaries or general beneficiaries qualifying for the PBS Safety Net were captured. In December 2004, the price of item 2481N increased from AUD$18.42 to AUD$29.84, exceeding the co-payment threshold of AUD$23.70. This transition to over co-payment resulted in more complete capture of medicine use; PBS-subsidised utilisation increased as a result, and under co-payment utilisation dropped off. As this medicine was above co-payment in 2012, it was not affected by the change in data collection by DHS.

**Fig. 4:**
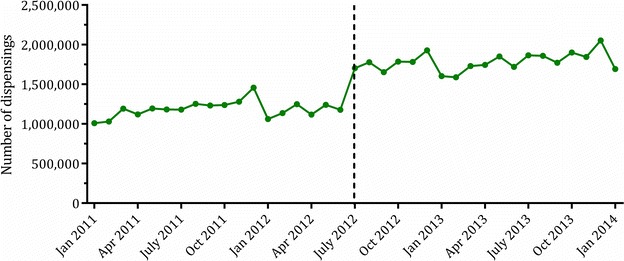
Utilisation of antidepressants increases in July 2012 with the addition of under co-payment data to the PBS dataset. Pharmaceutical Benefits Scheme and Repatriation Pharmaceutical Benefits Scheme Section 85 Supply data, Australian Government Department of Health [25]

However, the impact of this change on medicine ascertainment can be demonstrated by examining trends in antidepressant utilisation over this period. As many antidepressants are off-patent, there is a high rate of under co-payment utilisation of this class. Accordingly, Fig. 4 demonstrates a sharp increase in antidepressant prescriptions coinciding with the uptake of under co-payment medicines into the dataset.

The capture of under co-payment medicines has implications for analyses using unit-level data. If the medicine of interest is under co-payment for all or part of the study period, restriction of the study population to concessional beneficiaries or DVA clients can ensure more complete ascertainment of medicine use. This is because the concessional co-payment threshold is lower than the cost of any medicine on the PBS.

This method has been widely used in Australian studies using unit-level PBS data: of 113 such studies published between 1987 and 2013, 75 % employed a study population comprised of concessional beneficiaries or veterans [1]. As the concessional status of a patient can change over time, inclusion should ideally be further restricted to individuals for whom all dispensed medicines are provided at the concessional rate during the study period. Alternatively, under co-payment use of medicines requiring written or telephone authority approval can be tracked using the Authority Approvals database (such as use of dexamphetamine and methylphenidate in attention deficit hyperactivity disorder).

### Changes to medicine coding: ATC and PBS item code changes

Both PBS item and ATC codes are subject to change and this requires consideration when examining utilisation trends. For example, the antidepressant venlafaxine was listed under the ATC code N06AE06 until 1995, when its code changed to N06AA22. The code was further changed in 1999 to N06AX16 [33]. Similarly, a particular formulation of tobramycin, a systemic antibiotic, had the PBS item code 1356J until December 2005, when the code changed to 8872Y [34]. While the original item code still exists, it no longer refers to this formulation.

It is therefore important to ensure that all relevant historical and current ATC and/or PBS item codes are included in the analysis to avoid errors in utilisation estimates. Defining medicines of interest by ATC codes rather than item codes can help to overcome this problem, as ATC codes capture all current and historical PBS item codes, are less prone to change, and any historical changes in ATC code can be easily determined from the World Health Organisation Collaborating Centre for Drug Statistics Methodology (WHOCC) website [33]. One caveat is that there are occasional differences between the WHO-defined ATC codes and the ATC codes present in the PBS dataset. For example, lithium carbonate is classified as an antipsychotic by WHO (code N05AN01) but as an antidepressant by the PBS (code N06AX) [35]. Changes in PBS item codes are more difficult to track, but are recorded from 2003 in PBS monthly reports [36].

It is also worth noting that a medicine may concurrently have more than one ATC code (when the medicine has multiple indications affecting different body systems) or item code [according to the indication, strength, or prescriber (i.e. medical practitioner, nurse practitioner, or dentist) of a particular formulation]. Therefore, in some cases, the item code may provide a proxy of the indication or reason for prescribing. For example, the antineoplastic bevacizumab is available under item code 10114H for epithelial ovarian, fallopian tube or primary peritoneal cancer, but under 4400N for colorectal cancer. However, other drugs have multiple indications for prescribing combined under a single code (e.g. one item code for the antidepressant paroxetine is used for major depressive disorder, obsessive compulsive disorder, and panic disorder). Additionally, the validity of item codes for inferring patient diagnosis is uncertain. Depending on the research question, the researcher may choose to include all codes or only those referring to a certain indication or prescriber type.

### Policy changes

Changes in the medicine reimbursement process can impact data capture and estimates of utilisation. For example, the introduction of the Public Hospital Pharmaceutical Reforms from 2001 increased access to PBS-subsidised medicines by allowing participating public hospitals to provide PBS medicines to patients at discharge and outpatients. These Reforms are governed by individual agreements between each state and territory and the Australian Government. Agreements were initially established in Victoria (September 2001), followed by Queensland (August 2002), Western Australia (2002), Northern Territory (January 2007), South Australia (August 2008) and Tasmania (December 2010), with reforms implemented gradually across each state [37]. New South Wales and the Australian Capital Territory do not participate in the Reforms. Researchers conducting state-by-state comparisons should consider whether the introduction of the Reforms may influence utilisation estimates for the medicines of interest. A variable indicating the type of dispensing pharmacy (hospital, community) can be provided with PBS data by request.

In late 2011 the Pharmaceutical Reforms were varied to enable the introduction of a new scheme governing the subsidy of chemotherapeutic agents, the Revised Arrangements for the Efficient Funding of Chemotherapy measure [37]. These arrangements came into effect in December 2011 for private hospitals and community pharmacies, and April 2012 for public hospitals [38]. S100 medicines dispensed in public hospitals have traditionally been processed in bulk by DHS at the end of each month, and therefore were not recorded as individual-level dispensing claims or included in the dataset. However, the Efficient Funding of Chemotherapy resulted in a shift from bulk to unit-level processing of s100 chemotherapeutic items and increased capture of these medicines in the dataset. As such, an increase in utilisation of s100 medicines dispensed through public hospitals can be observed following the introduction of the scheme. These examples highlight the need to question significant and unexpected changes in the data to determine whether they represent a true change in utilisation or an artefact of the way the data are ascertained.

### Measures of utilisation

Medicine use can be quantified in a variety of ways in the PBS dataset, including by number of dispensings or costs. The strength of the medicine and quantity supplied can also be used to calculate DDD/1000 pop/day, a widely used measure of utilisation allowing for standardisation of drug use across countries and different forms of the drug. The DDD metric, established by the WHOCC, is based on the estimated mean daily dose of the drug when used for its main indication in adults [6]. DDD/1000 pop/day can be calculated for both plain products (which contain only one active ingredient) and combination products (with more than one active ingredient). The DUSC calculates DDD/1000 pop/day for combination products by counting the DDD for each constituent separately [7]; this method contrasts with that used by the WHOCC, who assign DDDs by counting the entire combination as one daily dose [39]. This methodological difference should be considered when making international comparisons of utilisation using DDDs.

Different measures of utilisation may yield differing results, and researchers must determine which measure(s) is most appropriate for their research question and dataset, considering the strengths and limitations of the chosen measure. Prescription-based measures such as ‘number of dispensings’ do not standardise utilisation across populations, or across different medicine strengths and pack sizes. While DDD/1000 pop/day is useful for standardising population-based measurements, the DDD on which it is based does not necessarily accurately reflect the dose recommended or prescribed. DDD is also limited for quantifying medicines use in children and the elderly, for whom different doses may be used. As with ATC codes, DDDs can change over time; a list of changes can be accessed from WHOCC [40].

**Fig. 5:**
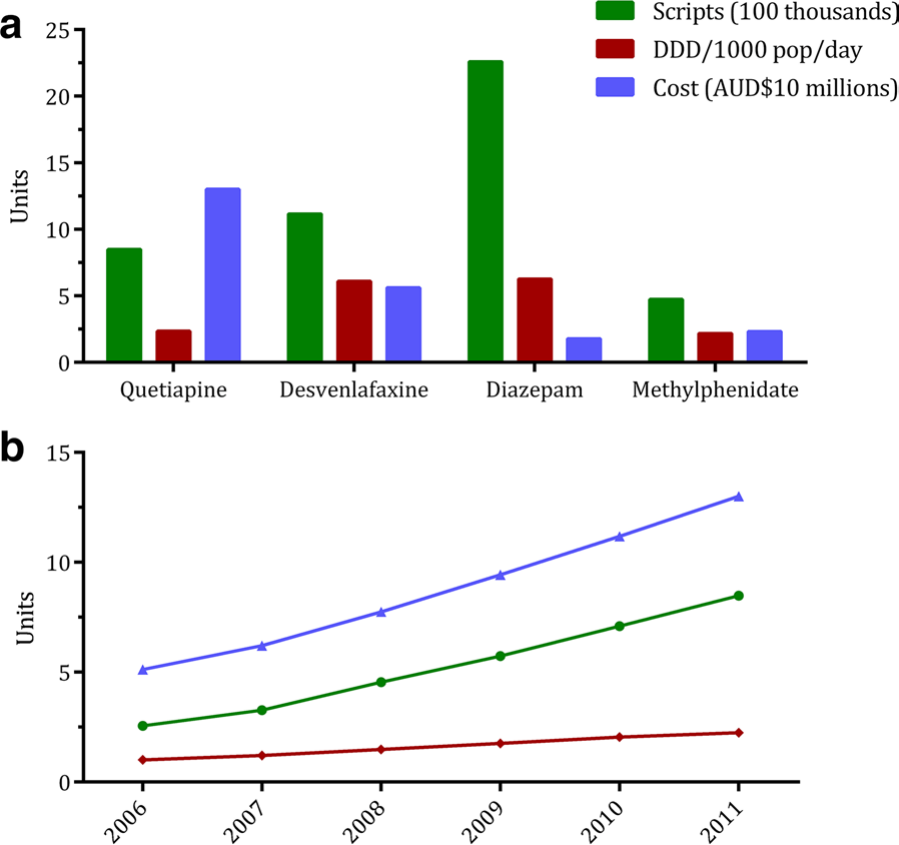
Comparison of utilisation according to prescriptions dispensed, DDD/1000 population/day, and cost to the Australian Government. **a** Relative use of four different psychotropic medicines in 2011, and **b** trends in the use of quetiapine from 2006 to 2011 depends on the measure of utilisation employed. Script and cost units represent 100 thousands of prescriptions and AUD$10 million, respectively. Australian Statistics on Medicines 2006–2011, Australian Government Department of Health [26]

Figure 5 demonstrates the differing results obtained when measuring utilisation by number of prescriptions dispensed, DDD/1000 pop/day, or medicine cost to government for the antipsychotic quetiapine; the antidepressant desvenlafaxine; the benzodiazepine diazepam; and the stimulant methylphenidate. Each of these measures have certain strengths and weaknesses. For example, the DDD of quetiapine is 400 mg, which is the average dose for an adult patient diagnosed with psychosis. However, a recent analysis by DUSC revealed that 23 % of patients taking quetiapine are using only the 25 mg strength of the drug, likely for the treatment of non-psychotic disorders such as anxiety and insomnia [41]; DDD/1000 pop/day would therefore likely under-estimate true use. In addition, quetiapine is one of the most costly medicines to the Australian Government [42], and analyses relying solely on cost would over-estimate its utilisation. Assessment of utilisation by number of prescriptions dispensed can also be problematic when comparing between different drugs, strengths and pack sizes. Quetiapine, for example, is usually dispensed in packs of 60 tablets, while desvenlafaxine has a pack size of 28. Use of different measures of utilisation may also impact trends in medicine use. For example, the increase in quetiapine use between 2006 and 2011 is more pronounced when measured by number of prescriptions (232 % increase) than by cost (154 % increase) or DDD/1000 pop/day (123 % increase).

## Conclusions

As a result of its universal healthcare arrangements, Australia has access to a whole-of-population dispensing database. The PBS data collection contains records on Australia’s 23 million citizens and all PBS, RPBS and under co-payment prescriptions, amounting to approximately 280 million dispensings a year [42]. The database is a valuable resource and has been widely used in both aggregated and unit-level analyses, and in linked and unlinked forms. The current paper has provided an overview of the PBS database and some of the extracts available to researchers, focussing primarily on publicly available, aggregated forms. Clearly these extracts hold great potential as a continuing source of data for pharmacoepidemiological research in Australia. However, the scope of the PBS database extends beyond the extracts and uses described herein.

We recently published a systematic review of all published literature using Australia’s PBS dispensing records between 1987 and 2013, identifying 228 studies using PBS data [1]. These studies explored a range of research questions, including trends in drug utilisation; clinical and patient practices around medicine use; drug use and outcomes; evaluations of interventions; and methodological studies undertaken using PBS claims. They also used a variety of PBS data extracts, including the publicly available Section 85 extract and PBS Statistics online, as well as a range of datasets available by request, such as the DUSC dataset, PBS 10 % sample, and DVA RPBS dataset. More than half of the studies combined PBS data with additional health data such as medical service claims—also under the custodianship of DHS—or other routine data such as hospitalisations, fact and cause of death data under the custodianship of Australia’s states and territories. Sixty-three studies linked person-level claims with other routine data collections, permitting exploration of drug safety and outcomes and the evaluation of the impact of interventions on utilisation. While the availability of linked data in Australia has traditionally been limited by legislative, privacy and cross-jurisdictional barriers, recent developments in data linkage infrastructure and governance in Australia bring the promise of increased access to population-based linked data for research purposes [43]. These developments, combined with the recent changes in data capture, such as the inclusion of under co-payment data in the PBS dataset from 2012, further strengthen the role of the PBS dataset in pharmacoepidemiological research and place Australia in a powerful position to conduct quality use of medicines research.

## Abbreviations

ASM: Australian Statistics on Medicines
ATC: Anatomical Therapeutic Chemical
Cth: Commonwealth
DDD: Defined daily dose
DDD/1000 pop/day: Defined daily dose per 1000 population per day
DHS: Department of Human Services
DoH: Department of Health
DUSC: Drug Utilisation Sub-Committee
DVA: Department of Veterans’ Affairs
FODD: Fact of Death Data
PBAC: Pharmaceutical Benefits Advisory Committee
PBS: Pharmaceutical Benefits Scheme
RCT: Randomised controlled trial
RPBS: Repatriation Pharmaceutical Benefits Scheme
S100: Section 100
S85: Section 85
TGA: Therapeutic Goods Administration
WHO: World Health Organisation
WHOCC: World Health Organisation Collaborating Centre for Drug Statistics Methodology
